# Quorum sensing in *Pseudomonas aeruginosa* mediated by RhlR is regulated by a small RNA PhrD

**DOI:** 10.1038/s41598-018-36488-9

**Published:** 2019-01-23

**Authors:** Anuja Malgaonkar, Mrinalini Nair

**Affiliations:** Department of Microbiology and Biotechnology Centre, The Maharaja Sayajirao University of Baroda, Vadodara, 390002 Gujarat India

## Abstract

*Pseudomonas aeruginosa* is a highly invasive human pathogen in spite of the absence of classical host specific virulence factors. Virulence factors regulated by quorum sensing (QS) in *P*. *aeruginosa* cause acute infections to shift to chronic diseases. Several small regulatory RNAs (sRNAs) mediate fine-tuning of bacterial responses to environmental signals and regulate quorum sensing. In this study, we show that the quorum sensing regulator RhlR is positively influenced upon over expression of the Hfq dependent small RNA PhrD in *Pseudomonas*. *RhlR* transcripts starting from two of the four different promoters have same sequence predicted to base pair with PhrD. Over expression of PhrD increased *RhlR* transcript levels and production of the biosurfactant rhamnolipid and the redox active pyocyanin pigment. A *rhlR::lacZ* translational fusion from one of the four promoters showed 2.5-fold higher expression and, a 9-fold increase in overall *rhlR* transcription was seen in the wild type when compared to the isogenic *phrD* disruption mutant. Expression, in an *E*. *coli* host background, of a *rhlR::lacZ* fusion in comparison to a construct that harboured a scrambled interaction region resulted in a 10-fold increase under *phrD* over expression. The interaction of *RhlR*-5′UTR with PhrD in *E*. *coli* indicated that this regulation could function without the involvement of any *Pseudomonas* specific proteins. Overall, this study demonstrates that PhrD has a positive effect on RhlR and its associated physiology in *P*. *aeruginosa*.

## Introduction

*Pseudomonas aeruginosa*, an opportunistic human pathogen, is the most important and common causative agent of chronic pulmonary infections in Cystic fibrosis (CF) patients^1^, infections in immunocompromised individuals having HIV or undergoing cancer chemotherapy and those with burn wounds^2^. Adaptive resistance towards antibiotics and decreased susceptibility of biofilms, protected by an extracellular matrix, make it difficult to eradicate *Pseudomonas* infections^3^. The gradual shift of an acute infection to a chronic disease is facilitated by virulence factors like elastase, rhamnolipids, lipopolysaccharide (LPS) and alginate^3,4^. Quorum sensing (QS) is a direct and major regulator of pathogenicity factors in *P*. *aeruginosa*, which gives a selective advantage to this pathogen over the host immune system by coordinating expression of several virulence genes^5^.

**Figure 1:**
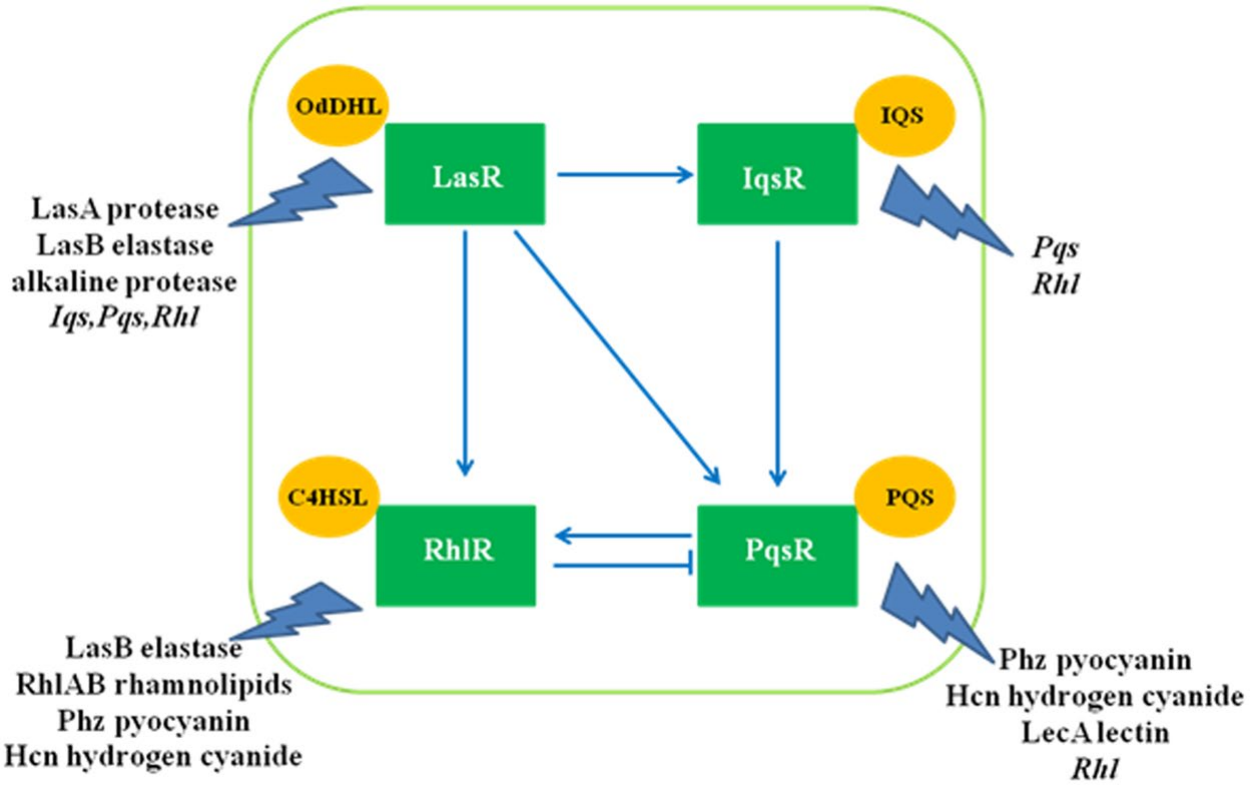
Quorum sensing (QS) in *P*. *aeruginosa*. A pictorial representation of the interconnected *las*, *rhl*, *pqs* and *iqs* systems and the virulence genes regulated by them. Concept courtesy^6^.

QS, in *P*. *aeruginosa*, is regulated in a hierarchical manner and consists of the interconnected *las*, *rhl*, *pqs* and *iqs* systems. The *las* system is at the apex of the QS circuit and its transcriptional regulator LasR in conjunction with its cognate autoinducer 3-oxo-C_12_-HSL induces the expression of *rhl*, *pqs* and *iqs* systems (Fig. 1)^6^, in a cell density dependent manner. The RhlR-C_4_-HSL complex is responsible for production of virulence factors like elastase B, rhamnolipid, hydrogen cyanide, lectins LecA and LecB, and pyocyanin either alone or in coordination with other branches of QS^4,6^.

Recent research on gene regulatory mechanisms have established small regulatory RNAs (sRNAs) as one of the major regulators of bacterial virulence^7^. Bacterial sRNAs range in size from 50–300 nucleotides, and function by affecting target mRNA translation and/or stability^8^. Base pairing between sRNA-mRNA occurs imperfectly and non-contiguously within a stretch of 6–25 nucleotides with the assistance of Sm-like, Hfq RNA chaperone protein^9^. Protein binding sRNAs sequester the RNA binding proteins which act as translational repressors or activators for several mRNAs^9^. The advent of techniques like RNA-Seq has lead to the identification of several sRNA candidates. However, only a few sRNAs have been characterized in *P*. *aeruginosa*^10–15^.

The work presented here provides the functional characterization of the small regulatory RNA PhrD as a significant regulator of RhlR mediated QS in *P*. *aeruginosa* under different conditions. The specificity of its regulation of RhlR is shown by means of transcriptional assays, reporter gene fusions and physiological assays in strains expressing altered levels of PhrD as well as studies in *E*. *coli* as a heterologous system.

## Results

### *In silico* analysis of PhrD

**Figure 2:**
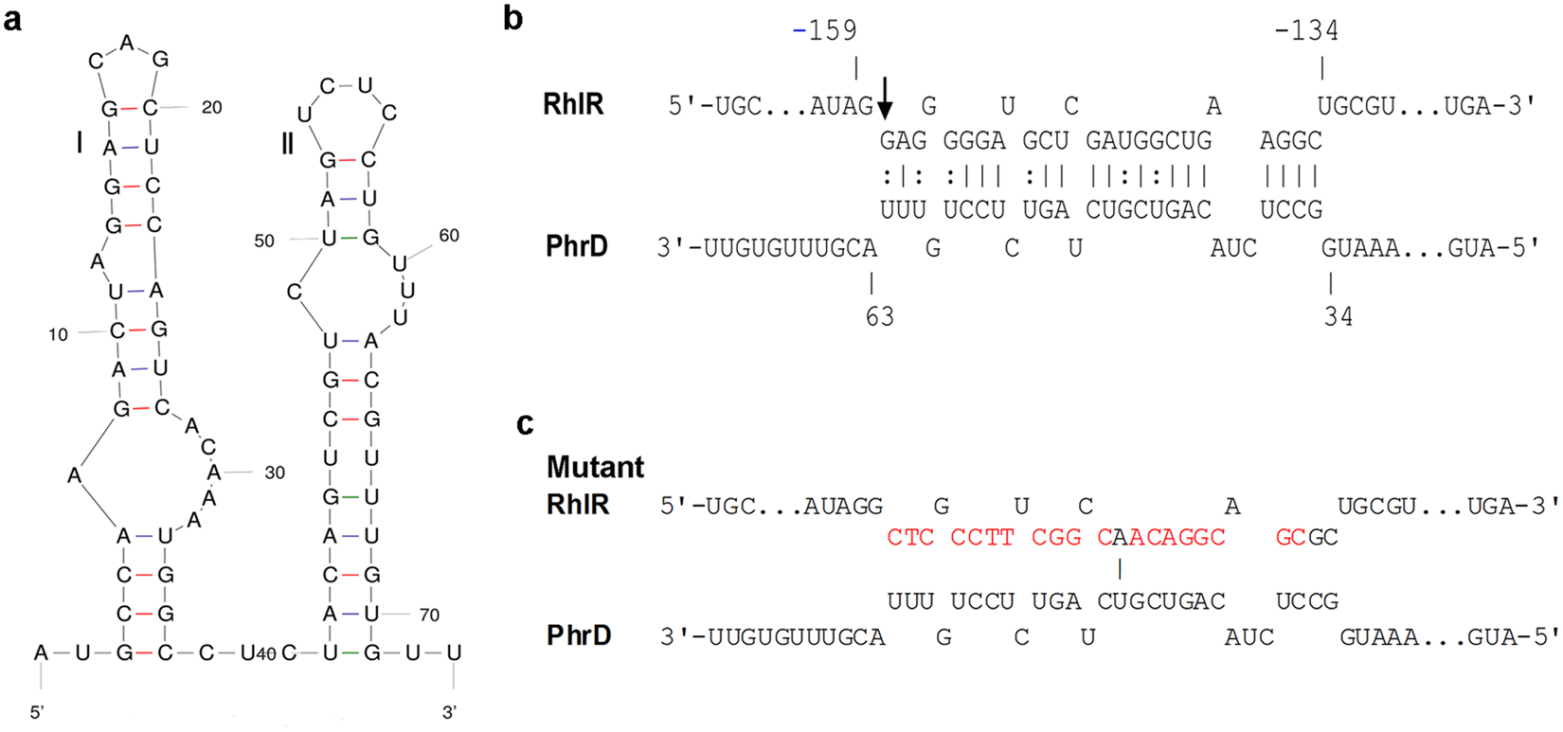
*In silico* analysis of PhrD. (**a**) Secondary structure of PhrD predicted by mfold. (**b**) RNA-RNA interaction between PhrD and *RhlR,* predicted using IntaRNA program. The arrow in the beginning of interaction region indicates the transcription start site of the P3 promoter of *rhlR*. The numbers on PhrD and *RhlR* are in reference to the nucleotides from the transcription start and the translational start respectively (**c**) Scrambled sequence of *RhlR*-PhrD interaction region incorporated in the mutant RhlR construct, B-pME6013.

**Figure 3:**
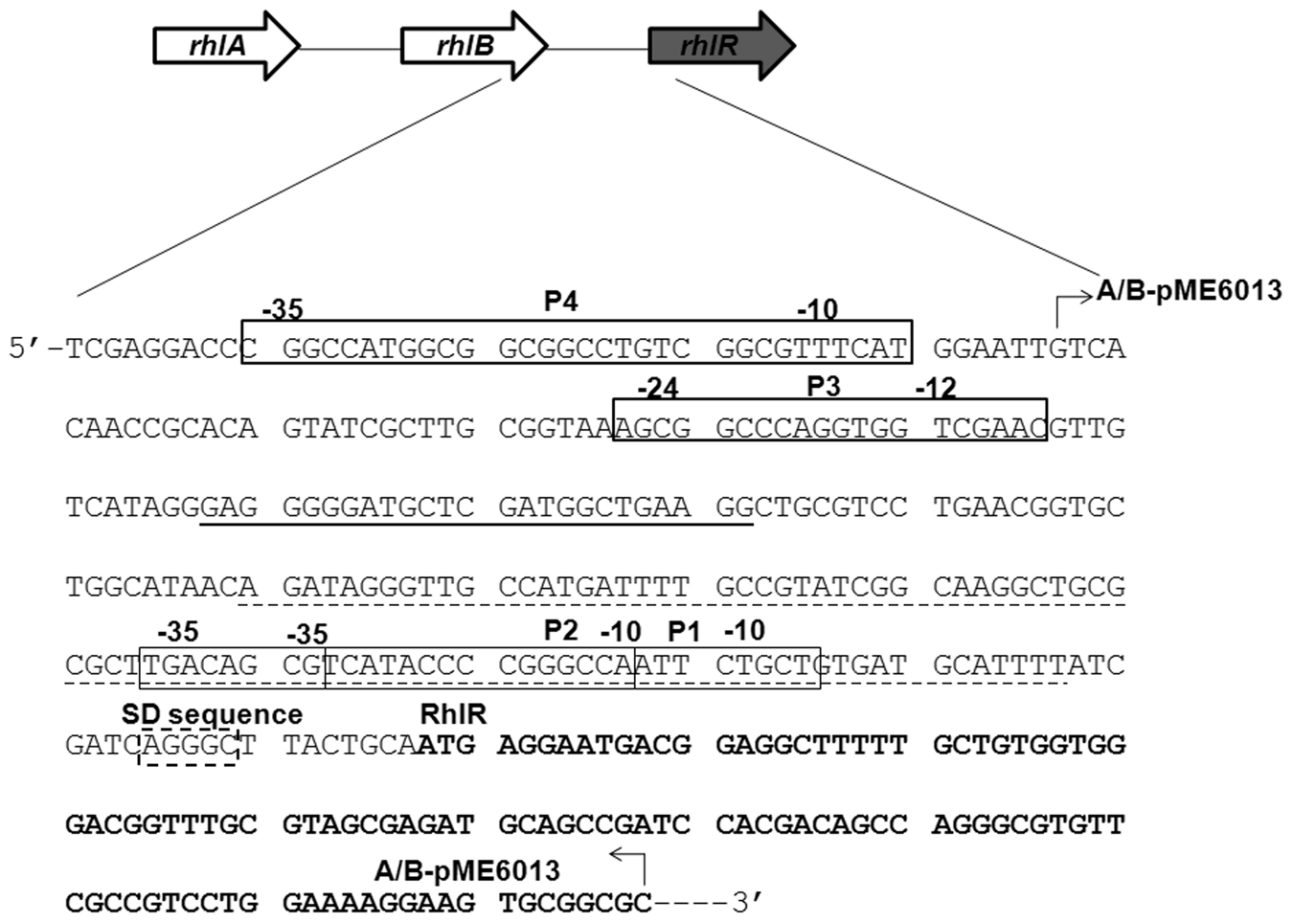
Schematic representation of promoter region of *rhlR* gene. The upstream region of *rhlR* gene comprises of four promoters, P1 to P4 that are indicated by boxes. The Shine Dalgarno (SD sequence) of *RhlR* is indicated by a dotted box and the first 33 codons of *RhlR* are in bold. The PhrD interaction region is underlined by a solid line. The region comprising the P1 and P2 promoters is excluded from the translational fusion and is shown by a dotted underline. A 141 bp region comprising the P3 promoter and the interaction region was amplified by PCR and fused to the 139 bp region comprising the SD sequence and the first 33 codons to get the *rhlR::lacZ* translational fusion in the constructs A-pME6013, and B-pME6013 (with scrambled interaction sequence).

PhrD, a 74 nt highly conserved small RNA^16^ was predicted to have two stem loops in its secondary structure (Fig. 2a). Putative targets of PhrD were predicted by RNA Predator program and confirmed by IntaRNA. A list of its first 50 putative targets can be found in Supplementary Table S1. Most of the predicted targets showed base pairing to the second stem (II) of PhrD. Out of the indicated targets, the major transcriptional activator of quorum sensing, RhlR, was chosen to study for its regulation by PhrD owing to its role in quorum sensing and regulation of pathogenicity genes^17^. *rhlR* gene reportedly has four promoters^17^ (Fig. 3), and the sequences coinciding with the transcription start site of the P3 promoter (downstream of P4) were predicted to bind with PhrD (−159 to −134, ‘G’ at position −158 being the transcription start site, Fig. 2b).

### Over expression and disruption of *phrD* and its levels under different nutrient conditions

**Figure 4:**
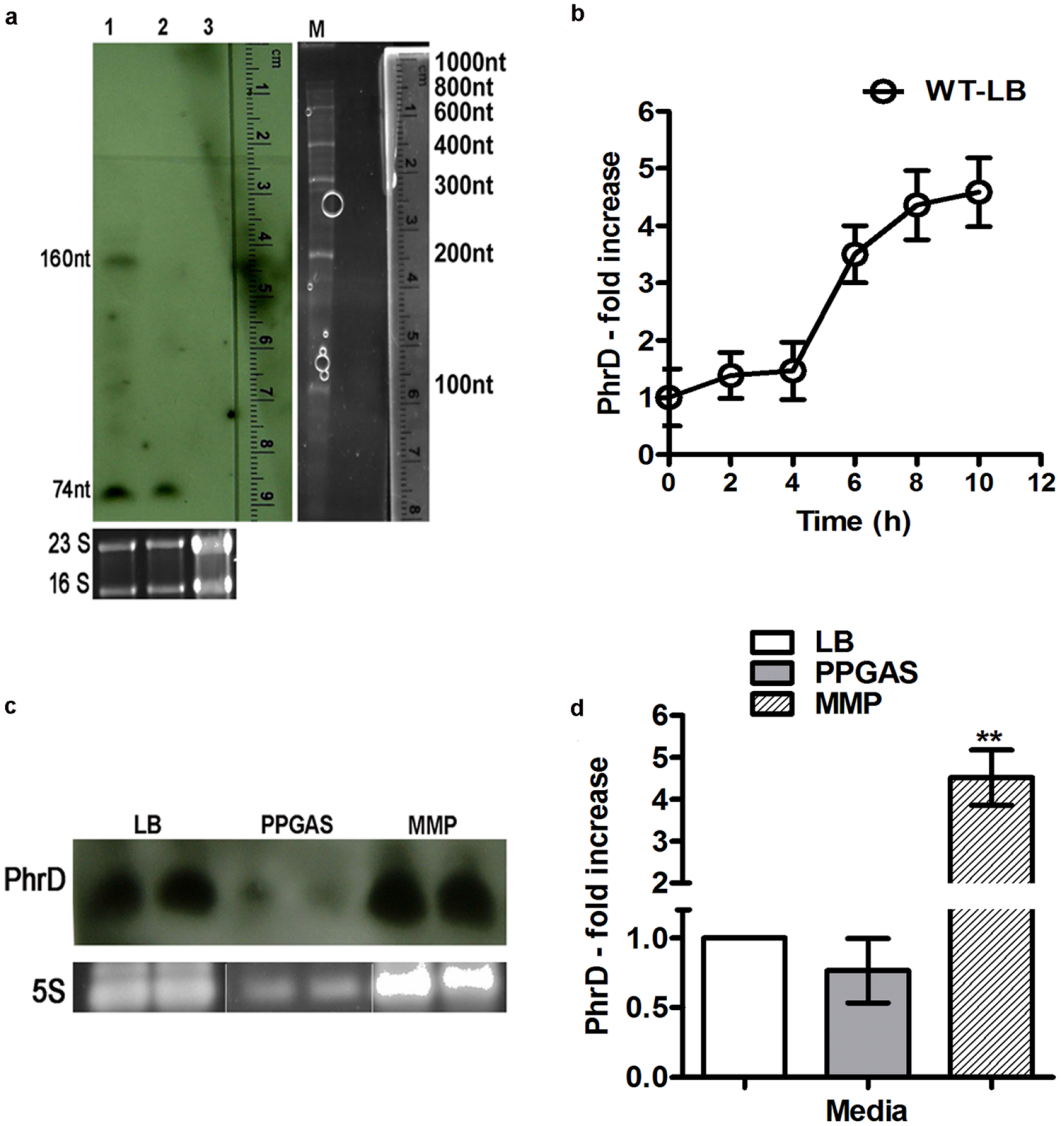
Expression analysis of *phrD* in *P*. *aeruginosa* strain PAO1. (**a**) Northern blots of PhrD in over expression and disruption. Lane 1-pHERD*phrD*: over expression; lane 2-pHERD30T: vector control; lane 3-*phrD*ΩGm: disruption strain. Ethidium bromide stained 23S and 16S rRNA bands on agarose gels are shown as loading controls. (**b**) Time course qRT-PCR of *phrD* from the WT in LB, normalized with 16S rRNA and 0 h expression as the calibrator. (**c**) *phrD* expression in the WT in LB, phosphate deficient PPGAS and nitrogen limited MMP media. 5S rRNA band on agarose gel is shown as the loading control. The gel has loading differences with the RNA in the PPGAS lane being poorly loaded. The two lanes of 5S rRNA used as loading control for PPGAS medium are cropped from a different gel and merged with the existing gel. See original gels in Supplementary Fig. S3. (**d**) Comparative PhrD levels in WT under nitrogen limited MMP and phosphate limited PPGAS media compared to that in Luria broth.

*phrD* without its promoter was over expressed in *Pseudomonas* under the *pBAD* promoter using the shuttle vector pHERD30T. Two prominent transcripts of 160 nt and 74 nt were expressed from the cloned plasmid and the chromosomal copy respectively in comparison with the control strain (Fig. 4a). The longer transcript derived from *phrD*, cloned downstream to the *pBAD* promoter was shown to base pair with *RhlR* transcript at the same nucleotides as would the chromosomal PhrD, as analyzed by IntaRNA program (Supplementary Fig. S1). Expression analysis of *phrD* in the wild type (WT) strain indicated a steady increase along the growth curve after normalization with 16S rRNA gene (Fig. 4b). Chromosomal *phrD* gene was disrupted by the introduction of a gentamicin resistance gene (Gm^R^) by homologous recombination. Disruption of *phrD* was confirmed by PCR with multiple primers (see Supplementary Fig. S2) and absence of transcript in northern blot (Fig. 4a, lane 3).

PhrD was predicted to base pair with *RhlR* mRNA, at the transcription start site of its σ^54^ (involved in N regulation) dependent P3 promoter which has been reported to express under phosphate limited conditions^17^. Expression analysis of *phrD* in Luria broth, nitrogen limited MMP and phosphate limited PPGAS medium showed that *phrD* expressed under all the studied conditions (Fig. 4c) with maximum levels in MMP (5–6 fold higher than the other two) (Fig. 4d).

### PhrD positively influences *rhlR* expression

**Figure 5:**
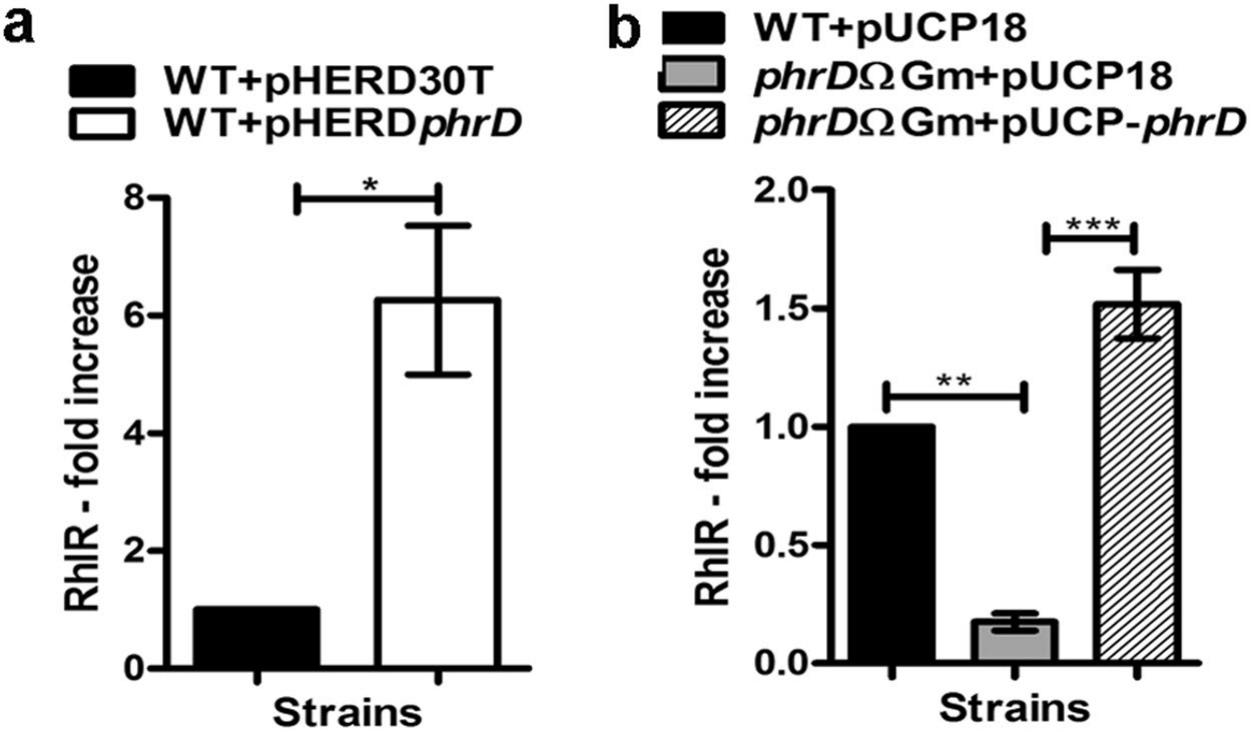
Effect of PhrD on *rhlR* expression. qRT-PCR analysis of *RhlR* transcripts from cells grown in Luria broth. Two different vectors (pHERD30T, pUCP18) bearing *phrD* were separately used for measurement of *RhlR* in WT background **(a),** and in complemented disruption **(b)**. (**a**) *Indicates statistically significant data between the strains determined by using Paired t test. (**b**) *Indicates statistically significant data between the strains determined by using one way ANOVA (**P < 0.01). The data is represented as the mean ± SD of three individual experiments and each sample was analyzed in triplicates.

The predicted interaction region of PhrD with *RhlR* coincides with the transcription start of P3 promoter of *rhlR* gene, at −159 to −134 bp upstream of the start codon of *RhlR* mRNA. *phrD* over expression in strain PAO1 influenced a 6-fold increase on *RhlR* in qRT-PCR assays (Fig. 5a). Disruption of *phrD* (*phrD*ΩGm) in PAO1, reduced *rhlR* expression to 0.2-fold while its complementation with pUCP-*phrD* restored the levels of *RhlR* (Fig. 5b).

**Figure 6:**
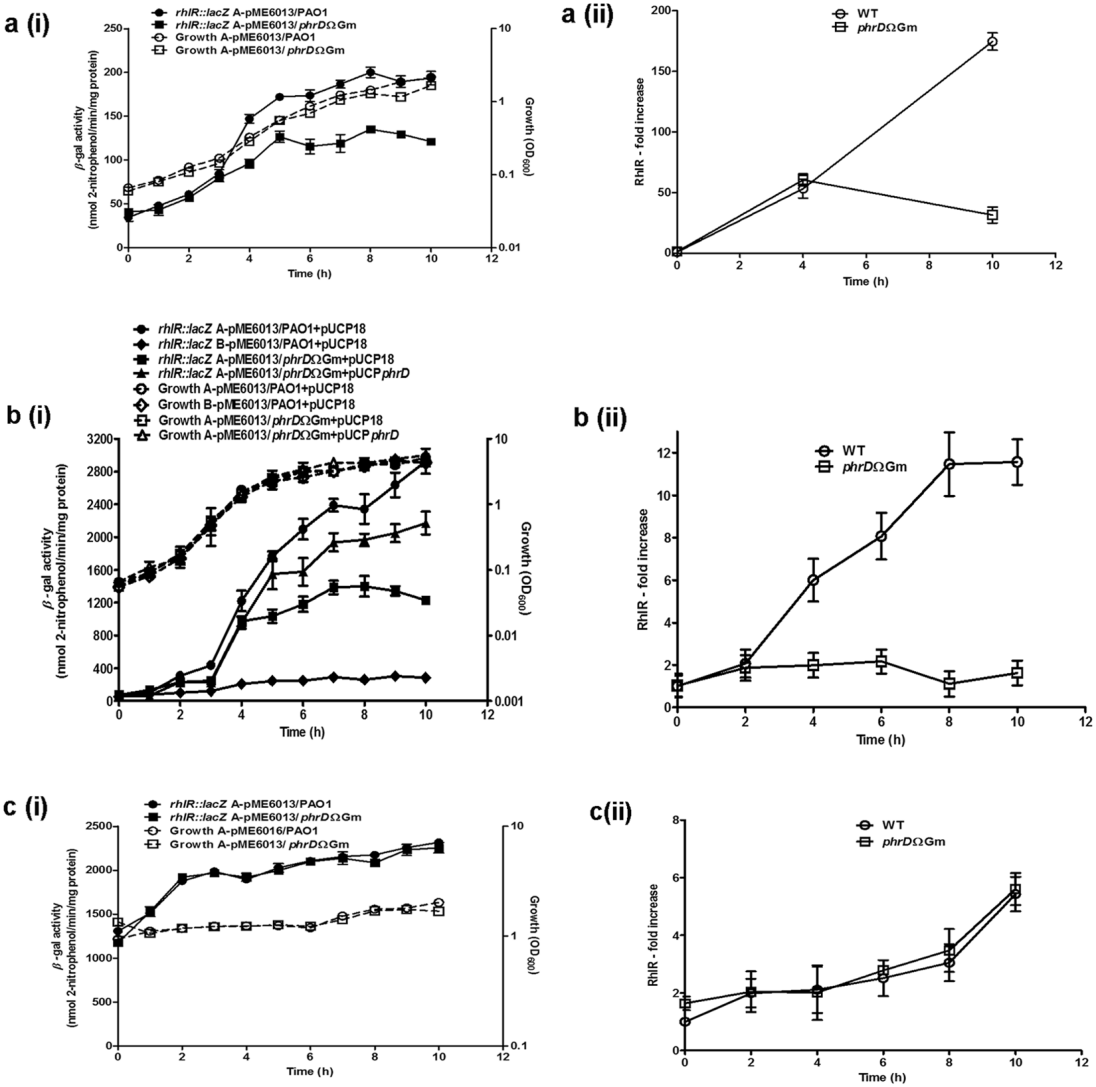
PhrD positively regulates *rhlR* expression in *P*. *aeruginosa* PAO1. Time course measurement of *RhlR* levels by *β-*galactosidase activity of P3 *rhlR::lacZ* fusion (i), and qRT-PCR (ii), measured in WT and *phrD* disruption mutant under: (**a**) Phosphate limited PPGAS medium (**b**) Luria broth; (**c**) N-limited MMP medium. In case of MMP, the cells were first grown to the desired OD in N-rich medium and resuspended in N-limited MMP. There was no net growth owing to nitrogen starvation. *β-*galactosidase activity of the disruption strain complemented with *phrD* over expression plasmid is measured in Luria broth. The data is represented as the mean ± SD of three individual experiments and each sample was analyzed in triplicates.

With an objective of establishing a correlation between PhrD and *RhlR* levels, *lacZ* reporter translational fusions were constructed from promoter P3-*rhlR* (Fig. 3 construct A-pME6013) and a time course measurement of *β*-galactosidase activity in PPGAS, LB and MMP media was correlated with *RhlR* transcript levels. *β*-galactosidase activity of the fusion exhibited a specific increase along the log phase up to 10 h in the WT and the disruption strains. The increase in WT, as compared to the disruption, was 1.5 and 2.5-fold in PPGAS and LB respectively (Fig. 6a(i), b(i)). This paralleled with a 6–9 fold increase in the overall transcript levels of *RhlR* in the WT PAO1 with respect to the disruption, in these media (Fig. 6a(ii), b(ii)). Comparing the increase observed in qRT-PCR with that of P3-*rhlR::lacZ* fusion explains that the difference in the fold increase might arise as a consequence of the modulation of transcripts arising from P4 promoter also.

Complementation of the disruption strain by the cloned PhrD restored its *β*-galactosidase levels to ~75% of that of the WT (Fig. 6b(i)). Expression of B-pME6013 bearing a non-specific scrambled interaction sequence did not show any increase even in the WT (*phrD*^+^) background (Fig. 6b(i)). These results corroborated the positive influence of PhrD with a sequence specific interaction to *RhlR* transcripts.

The expression of P3-*rhlR::lacZ* was very high and maximum in nitrogen limited MMP medium as compared to other media, and had no difference between WT and disruption strains (Fig. 6c(i)). This result perhaps indicated that under amino acid starvation there is an involvement of stringent response mediated by ppGpp in the high expression of *rhlR*^18,19^, alleviating the requirement of PhrD under these conditions.

### RhlR expression is regulated by PhrD in a heterologous system

**Figure 7:**
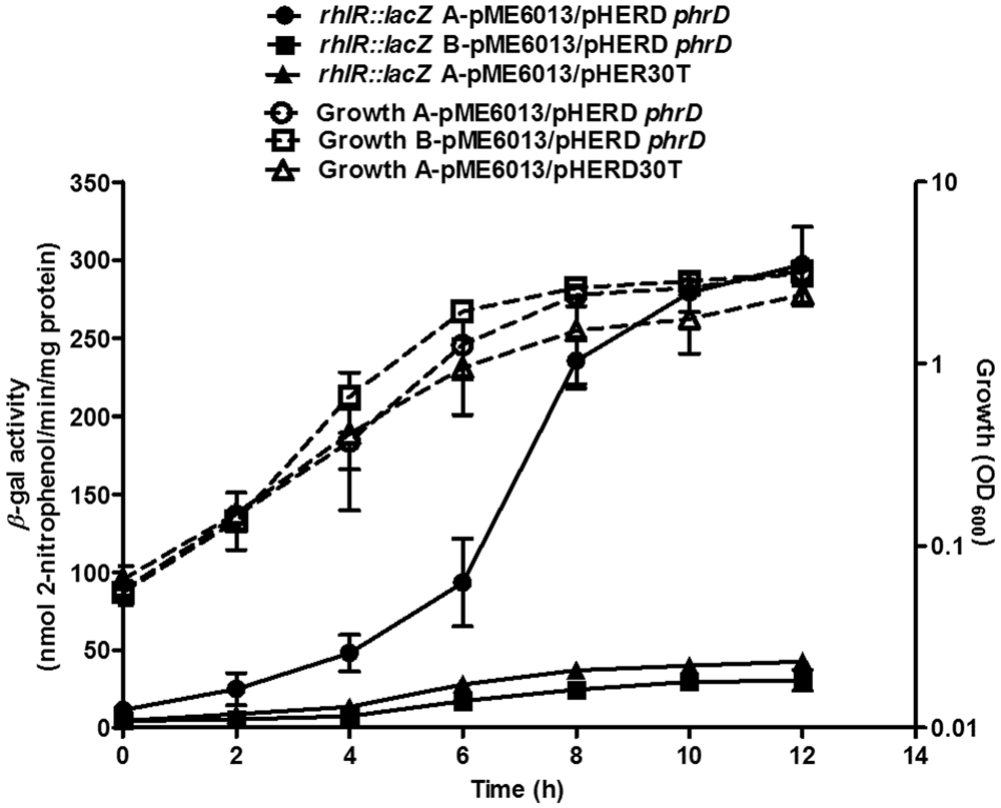
PhrD regulates *rhlR* in a heterologous system. *β*-galactosidase activities from *rhlR::lacZ* fusion plasmids (continuous lines, filled symbols) and growth (dotted lines, open symbols) in *E*. *coli* cultures growing in LB at different time points. A-pME6013- fusion bearing intact PhrD interaction region; B-pME6013- fusion with scrambled interaction region; pHERD*phrD*- *phrD* over expression plasmid. The results are representative of three individual experiments.

Subsequent to the positive influence of PhrD on *rhlR* expression, the specificity of their interaction was assayed in the heterologous host *E*. *coli* with the constructs A-pME6013 and B-pME6013. A-pME6013 harboured the intact interaction region (Fig. 2b) whereas B-pME6013 contained a scrambled stretch of the same 25 nucleotides (Fig. 2c) instead of the predicted interaction region and served as a negative control. When introduced into an *E*. *coli* containing the PhrD over expressing plasmid, *β*-galactosidase activity of the fusion with intact interaction region showed a specific increase of 10-fold with growth (Fig. 7). No significant increase was observed in the expression of P3-*rhlR::lacZ* fusion with the scrambled PhrD-*RhlR* mRNA interaction region, and in A-pME6013 with vector plasmid (no PhrD). These results proved the specific involvement and significance of base pairing interaction of PhrD with *RhlR* mRNA at the stretch of 25 nucleotides at the 5′UTR. Also, regulation of *RhlR* by PhrD in a heterologous system like *E*. *coli* proved that the interaction between PhrD and *RhlR* could be carried out without the assistance of any *Pseudomonas* specific proteins.

### PhrD positively influences rhamnolipid and pyocyanin production

**Figure 8:**
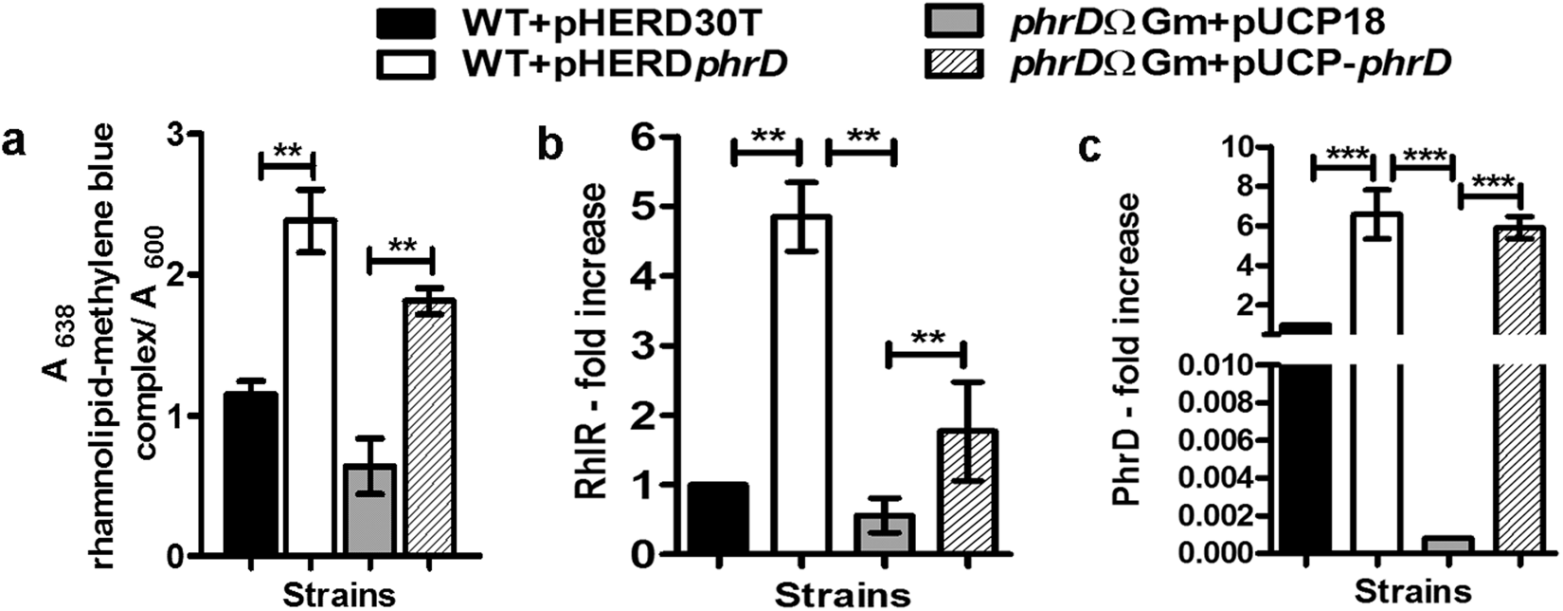
PhrD positively influences rhamnolipid production (**a**) Spectrophotometric assay for rhamnolipid measurement from culture supernatants by methylene blue method. Increase in rhamnolipid production is represented as increase in the absorbance at 638 nm normalized to absorbance at 600 nm of the culture. (**b**,**c**) *RhlR* and *PhrD* levels respectively. All these measurements were carried out in M9 medium. **Indicates statistically significant data at P < 0.01 as analyzed by one way ANOVA. Each experiment was performed thrice.

**Figure 9:**
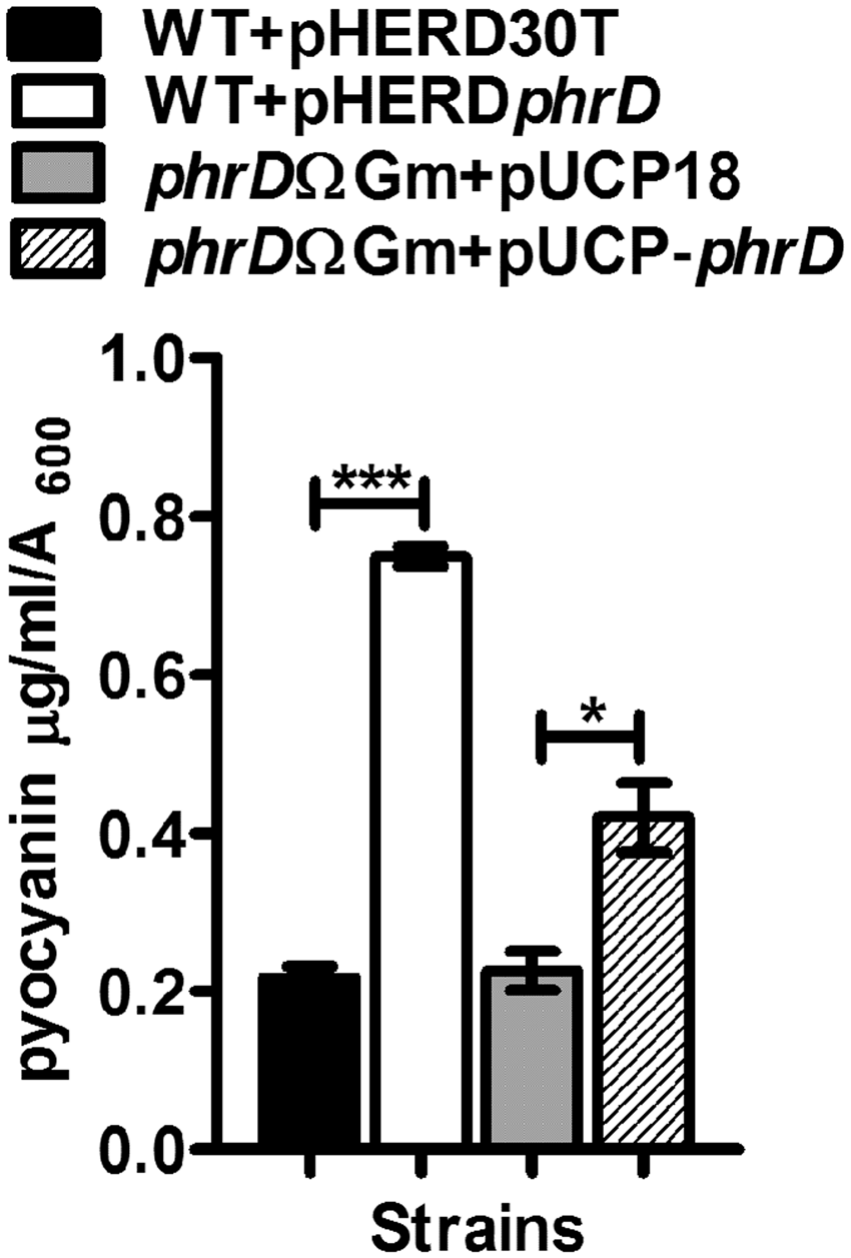
Pyocyanin production under altered levels of PhrD. Pyocyanin levels in LB were measured as µg/ml/OD_600_ of the culture. **and *indicate statistically significant data at P < 0.01 and P < 0.05, as analyzed by one way ANOVA performed on data obtained from three independent experiments.

RhlR, in complex with its cognate autoinducer C_4_-HSL, stimulates the synthesis of important virulence factors like rhamnolipids and pyocyanin pigment^6^. Therefore, an effect on their production, in relation to PhrD could be partially attributed to the regulation of *rhlR* by this small RNA. Rhamnolipids are known to be produced maximally under elevated C/N ratio under nitrogen exhaustion^20^. Measurement of rhamnolipid levels in M9 defined medium with 0.4% glycerol and low nitrogen source (0.1 g/L KNO_3_) showed an increase of 2.5-fold under *phrD* over expression (Fig. 8a). The disruption strain that showed 0.5-fold level of rhamnolipid was restored to 1.8-fold when complemented with multi-copy PhrD. The rhamnolipid levels in these strains are in correlation with that of *RhlR* and PhrD levels in M9 (Fig. 8b,c). Likewise, the pyocyanin production in LB that was increased by 4-fold in pHERD*phrD* strain is also in correlation with the corresponding *RhlR* levels (Figs 9 and 5a).

## Discussion

This paper reports the functional characterization of a sRNA PhrD and its influence on quorum sensing in *P*. *aeruginosa* under different nutrient conditions that mimic host physiology in pathogenesis. PhrD was shown to base pair at the transcription start site of the σ^54^ dependent P3 promoter of *rhlR* (downstream of P4) which was reported to express under phosphate limited PPGAS medium^17^. A steady increase in the transcript levels of *RhlR* in the presence of PhrD indicated its role in modulation of *rhlR* expression from P3 and P4 promoters (Fig. 6a(ii), b(ii)). Enhancement of expression of P3-*rhlR::lacZ* fusion by PhrD, observed in PPGAS and Luria broth (Fig. 6a(i),b(i)), signifies its role in regulation of *rhlR* expression under phosphate deficient and nutrient rich conditions. Although *rhlR* expression is reported to be dependent on LasR when grown in Luria broth^21^, our results indicate that PhrD is an additional regulator of RhlR under these conditions.

PhrD had no influence on *rhlR* expression in MMP medium (Fig. 6c(i), c(ii)), its role perhaps being overruled by stringent response under this condition. Amino acid starvation in *P*. *aeruginosa*, promptly elicits the RelA protein mediated stringent response (SR) that synthesizes the signalling molecule (p)ppGpp. The QS-based response is mediated by RelA, since a (p)ppGpp-null SR mutant (*spoT relA*) shows reduced *rhlI*, *rhlR*, *lasI*, and *lasR* expression^19^. Further, overexpression of RelA leads to the early transcription of the *lasR* and *rhlR* genes, production of QS signals, and overproduction of QS-dependent virulence factors rhamnolipids, pyocyanin, elastase *etc*.^18^

The positive influence of PhrD on the expression of *rhlR::lacZ* fusion observed in *E*. *coli* host background proves the specific interaction between *RhlR* and PhrD without the requirement of *P*. *aeruginosa* specific proteins (Fig. 7). This interaction was shown to be sequence specific, and a direct base pairing between PhrD sRNA and *RhlR* mRNA manifested this regulation. The *Pseudomonas* background however facilitated better expression of *rhlR* than by *E*. *coli,* probably owing to better recognition of P3 promoter by *Pseudomonas* sigma factors or involvement of additional *Pseudomonas* proteins. A similar heterologous *E*. *coli* host system was used earlier for *in vivo* identification of nucleotides important for regulation of *HapR* mRNA by Qrr sRNA of *V*. *cholerae*^22^.

The increase induced by PhrD in the production of rhamnolipid and pyocyanin mediated by RhlR, reflected an indirect regulation of these pathogenicity factors by this sRNA (Figs 8a and 9). The haemolytic activity as well as high surface activities of rhamnolipids cause lyses of polymorphonuclear leukocytes and monocyte-derived macrophages leading to necrosis^3^. Strains lacking in rhamnolipid production are rapidly cleared from the lungs of *Pseudomonas* infected mice^23^. Pyocyanin influences pathogenicity by causing goblet cell metaplasia and hyperplasia, airway fibrosis, and alveolar airway destruction in cystic fibrosis lungs^24^. It damages human cells by causing inhibition of cellular respiration, ciliary function, epidermal cell growth, prostacyclin release and disruption of calcium homeostasis^2^. PhrD-directed increase in the synthesis of these RhlR associated virulence factors may enhance invasiveness and colonization of the pathogen.

PhrD regulates quorum sensing under different conditions in a generalized manner in *P*. *aeruginosa* unlike other previously characterized sRNAs: RsmY regulates *rhl* system under nutrient rich conditions^11^. PhrS activates PqsR quorum sensing regulator under oxygen limited conditions leading to increased pyocyanin production^13^. Base pairing of NrsZ sRNA with *rhlA* activates rhamnolipid production under nitrogen limitation^14^. Our findings show that sRNA PhrD enhances both, rhamnolipid and pyocyanin production, by positively influencing RhlR.

This work establishes a substantial route of positive regulation of *RhlR* transcripts generated from multiple promoters upstream to the PhrD interaction region. PhrD thus proves out to be a sRNA that assists this bacterium to tune in to the environmental and host induced stimuli.

## Methods

### Bacterial strains, plasmids and growth conditions

The strains and plasmids used in the study are mentioned in Table 1. For routine culturing, bacterial cultures were grown in Luria broth (HiMedia, Mumbai, India) at 37 °C and supplemented with antibiotics as and when required. All the antibiotics were bought from HiMedia, Mumbai, India and were added to a final concentration of 30 µg/ml of gentamicin(Gm), 300 µg/ml of carbenicillin(Cb), 100 µg/ml of tetracycline for *P*. *aeruginosa* and 10 µg/ml of gentamicin, 100 µg/ml of ampicillin(Ap) and 25 µg/ml of tetracycline(Tc) for *E*. *coli*. For rhamnolipid measurements, cultures were grown in M9 minimal medium (1X M9 salts, 2 mM MgSO_4_, 0.4% glucose, 100 µM CaCl_2_). 5X M9 salts contained Na_2_HPO_4_.7H_2_O 64 g/L, KH_2_PO_4_ 15 g/L, NaCl 2.5 g/L, NH_4_Cl 5 g/L^25^. For *β*-galactosidase assays, cultures were grown in Minimal medium P (MMP: 20 mM glucose, 0.1% casamino acids, Na_2_HPO_4_ 1.47 g/L, KH_2_PO_4_ 0.648 g/L, MgSO_4_ 0.2 g/L, FeSO_4_ 0.001 g/L^26^ or phosphate limited peptone/glucose/ammonium salts medium (PPGAS: 0.5% glucose (w/v), 1% Peptone (w/v), NH_4_Cl 20 mM, KCl 20 mM, Tris/HCl 120 mM pH 7.2, MgSO_4_ 1.6 mM)^17^ or Luria broth. Cells grown to logarithmic phase in N-rich medium were shifted down to N-limited MMP for the *β*-galactosidase assay.

**Table 1:** Bacterial strains and plasmids used in the study.

Strains/Plasmids	Relevant features	Reference/Source
**Strains**		
*E. coli* DH5α	*recA1 endA1 hsdR17 thi-1 supE44 gyrA96 relA1 deoR* Δ(*lacZYA-argF*)*U169 *(Φ80*lacZ*ΔM15)	Lab stock
*P. aeruginosa* PAO1	Wild type	^37^
*phrD*ΩGm	PAO1 strain with disrupted *phrD*; Gm^r^	This study
**Plasmids**		
pBluescriptKS(+)	Cloning vector ColE1 replicon;Amp^r^	Lab stock
pHERD30T	*E. coli-Pseudomonas* shuttle vector carrying inducible *pBAD* promoter; Gm^r^	Paolo Visca, Roma Tre University, Rome, Italy
pHERD*phrD*	pHERD30T with *phrD* gene	This study
pUCP18	*Escherichia-Pseudomonas* shuttle vector; Cb^r^	^38^
pUCP-*phrD*	pUCP18 with *phrD* gene	This study
pUCP18-RedS	λ recombinase vector; Ap^r^	^32^
pBBRMCS5	Broad host range cloning vector; Gm^r^	^39^
pphrD-GDC	pTZ57R with *phrD* disrupted by Gm^R^; Ap^r^	This study
pME6013	Translational *lacZ* fusion vector; Tc^r^	Elisabeth Sonnleitner, Max. F. Perutz Laboratories, Vienna, Austria
A-pME6013	*rhlR::lacZ* translational fusion with intact PhrD interaction region in pME6013	This study
B-pME6013	*rhlR::lacZ* translational fusion with scrambled PhrD interaction region in pME6013	This study

### Bioinformatics

The sequence of PhrD sRNA was retrieved from Pseudomonas Genome database^27^ and its secondary structure was determined using the mfold Web Server^28^. RNA Predator^29^ and IntaRNA program^30^ were used to predict the putative targets of PhrD.

### Construction of plasmids

All the cloning procedures were carried out as per the standard molecular biology protocols^25^. A 134 bp region containing the *phrD* gene without its endogenous promoter was amplified with primer pair PhrDF and PhrDR (Table 2) from genomic DNA of PAO1 and cloned in *E*. *coli-Pseudomonas* shuttle vector pHERD30T under arabinose inducible *pBAD* promoter at *Xba*I-*Pst*I sites. The recombinant plasmid was electroporated into *Pseudomonas*, to give pHERD*phrD*, using the protocol mentioned elsewhere^31^.

**Table 2:** Primers used in the study.

Primer name	Sequence	Specification
**Cloning**		
PhrDF	GC**TCTAGA**ATGCCAAGACTAGGAGCAG	Fwd primer
PhrDR	TGCA**CTGCAG**AGCGGGGATTTACTATTTGT	Rev primer
FupPhrD	GATCCGGGAGCGAACC	Upstream of PhrD
RupPhrD-Gm	CCGTTTCCACGGTGTGCGTCGCCATTTGTGACTGGAGCTG	Upstream of PhrD
FdnPhrD-Gm	GTAAATTGTCACAACGCCGCCAGTCGTCTAGTCTCCTGTTTACG	Downstream of PhrD
RdnPhrD	TCCCTTAAATCAGTTTGAAAATAAAA	Downstream of PhrD
GmF	GACGCACACCGTGGAAAC	Fwd primer, Gm
GmR	CGGCGTTGTGACAATTTACC	Rev primer, Gm
AM_101	CG**GAATTC**GTCACAACCGCACAGTATCG	Fwd primer, P3
AM_102	TGCAGTAAGCCCTGATCGATGTTATGCCAGCACCGTTCAG	Rev primer, P3
AM_103	ATCGATCAGGGCTTACTGCA	Fwd primer:RhlR RBS → 33 codons,
AM_104	AA**CTGCAG**GCGCCGCACTTCCTTTTC	Rev primer:RhlR RBS → 33 codons.
AM_111	GCTGCCTGTTGGCCGAAAGGCGAGCCTATGACAACGTTCGACCA	Rev primer: P3 with scrambled sequence
AM_112	CTCGCCTTTCGGCCAACAGGCAGCGCTGCGTCCTGAACGGTGCTGGCATAACATCGATCAGGGCTTACTGCA	Fwd primer: RBS with scrambled interaction sequence
**qRT-PCR**		
PhrD RTF	AGCAGCTCCAGTCACAAATG	
PhrD RT R	CAAACGTAAACAGGAGACTAGACG	
RhlR F	CTGGGCTTCGATTACTACGC	
RhlR R	CCCGTAGTTCTGCATCTGGT	
16S F	CTCAGACACAGGTGCTGCAT	
16S R	CACCGGCAGTCTCCTTAGAG	

Regions in bold are restriction sites, Gm- gentamicin, Underlined regions are overlapping regions used for fusion PCRs.

### Construction of *rhlR::lacZ* translational fusion

In order to create *rhlR::lacZ* translational fusions, a 141 bp long sequence that includes the P3 promoter and the PhrD interaction region of *RhlR* was amplified (primer pair AM_101 and AM_102) and fused to a 139 bp long 5′UTR region with RBS and the first 33 codons of *RhlR* mRNA (primers AM_103 and 104), by overlap extension PCR (Fig. 3). The PCR fragment was fused to the eighth codon of *lacZ* at *Eco*RI*-Pst*I digested pME6013 to get translational fusion of *rhlR* with *lacZ*, A-pME6013. A similar construct B-pME6013 that served as a negative control was made where in the interaction region was substituted with a commercially synthesized scrambled sequence (primers AM_111 and AM_112). Neither of these constructs included the P4, P2 and P1 promoters mentioned by Medina and colleagues^17^.

### Construction of *phrD* disruption mutant

*phrD* gene was disrupted by gentamycin resistance marker, cloned into plasmid pTZ57R/T and transformed into PAO1 strain to achieve disruption of chromosomal *phrD* by homologous recombination. An 855 bp long gentamicin marker was amplified with GmF and GmR primer pair using pBBRMCS5 plasmid as the template. The 734 bp and 792 bp long upstream and downstream regions consisting of 5′ and 3′ ends of *phrD* respectively were amplified using primers FupPhrD and RupPhrD-Gm and FdnPhrD-Gm and RdnPhrD respectively from genomic DNA of strain PAO1. The reverse and forward primers of the above primer pairs shared a 20 bp homology with the gentamicin marker. An overlap extension PCR was done to fuse the above amplified fragments and the resulting gene disruption cassette of *phrD* was cloned in pTZ57R/T plasmid using TA overhangs. The disrupted gene in the recombinant plasmid was recombined into strain PAO1 containing the recombinant proficient vector pUCP18-RedS expressing the λ red recombinase^32^. Gentamicin resistant, carbenicillin sensitive colonies were screened with PCR to confirm the chromosomal disruption of *phrD* (Supporting gels in Supplementary Fig. S2).

For complementation of *phrD* disruption mutant, a 146 bp long *Xba*I-*Pst*I released fragment from pHERD*phrD*(Gm^r^) was sub-cloned into plasmid pUCP18(Cb^r^) under *lac* promoter and transformed into gentamicin resistant disruption strain. Complementation of *phrD*ΩGm was achieved by expressing *phrD* from pUCP18.

### RNA isolation and Northern Blot

Total RNA was extracted from stationary phase cultures grown in different media by acid guanidinium thiocyanate–phenol–chloroform method^33^ for Northern Blot, and Roche High Pure RNA purification kit for real time PCR, as per manufacturer’s instructions. For northern blots, total RNA was separated on 6% polyacrylamide/6 M urea gels and electroblotted on to the nylon membrane. The RNA was UV cross-linked to the membrane followed by hybridization with PhrD probe, labelled by Digoxygenin-dUTP using DIG-labeling kit as per manufacturer’s instructions (Roche, USA). DNA fragment released with *Xba*I-*Pst*I digestion from pHERD*phrD* was used as a template for PhrD probe preparation. The hybridized probes were immunodetected using anti-digoxygenin-AP, Fab fragments and visualized with the chemiluminescence substrate CSPD (Roche, USA) on X-ray films.

### Real time PCR assays

Real time PCR assays were performed using total RNA isolated from cultures grown in Luria broth to an OD_600_ of 2 as per the manufacturer’s instructions (High Pure RNA isolation kit, Roche, Basel, Switzerland) to study the effect of altered levels of PhrD on *rhlR* expression. For expression analysis of PhrD under nitrogen limited or phosphate deficient conditions, RNA was extracted from cells grown to an OD_600_ of 1.5 in MMP and PPGAS media respectively. Influence of PhrD on *rhlR* expression was studied by performing qRT-PCR. RNA was extracted from cells harvested every 2 h from WT and *phrD*ΩGm cultures grown in Luria broth, PPGAS and MMP.

cDNA first strand synthesis was done using Genei RT-PCR kit (Bangalore Genei, Bangalore, India). Real time PCR was performed using Maxima SYBR Green/ Rox qPCR Master Mix (2×) (Thermo Scientific, Massachusetts, USA) and fold expression was determined after normalization with 16S rRNA gene by 2^−ΔΔCt^ method^34^. The WT strain consisting of empty vector pHERD30T was used as the calibrator for *rhlR* expression analysis whereas expression of PhrD in MMP and PPGAS medium was compared to that in Luria broth.

### Rhamnolipid measurement

Culture supernatant from bacterial cultures grown in M9 minimal media for 24 h was adjusted to pH 2.5 ± 0.2 using 1 N HCl. The acidified sample was then extracted with 5 volumes of chloroform. 4 ml of chloroform extract was allowed to react with freshly prepared methylene blue solution containing 200 µl of 1 g/L of methylene blue (prepared in 10 mM borax buffer pH 10.5 and stabilized by adjusting pH to 5.5) and 4.9 ml of distilled water. The samples were mixed vigorously and allowed to stand. The absorbance of the chloroform phase was read at 638 nm with chloroform as blank with Beckman Coulter DU^®^ 720 spectrophotometer. The absorbance values were normalized with A_600_ of the cultures^35^. Each sample was analyzed in triplicates and results are average of three independent experiments.

### Pyocyanin assay

5 ml of stationary phase grown cultures in Luria broth were extracted with 3 ml chloroform. The pyocyanin containing chloroform layer was acidified with 0.1 N HCl and absorbance of the pink colored acid fraction was measured at 520 nm. Pyocyanin levels were expressed as µg/ml/A_600_ of culture supernatant^13^. The experiment was done thrice in triplicates.

### *β*-galactosidase assay

200 µl cultures were periodically withdrawn from cells growing in Luria broth, MMP or PPGAS media. *β*-galactosidase activity was determined as described previously after normalization with protein in mg^36^. The Miller units are represented as mean of three independent experiments.

### Statistical analyses

All the experiments were performed in triplicates and performed thrice (n = 3). Data analyses was carried out by either Paired t test or one way analysis of variance (ANOVA) followed by Tukey’s multiple comparison test using Graph Pad Prism 6.0 (CA, USA).

## Electronic supplementary material

Supplementary information
