# Supplementary material for: Quorum sensing in Pseudomonas aeruginosa mediated by RhlR is regulated by a small RNA PhrD
Source: Sci Rep. 2019 Jan 23;9:432. doi: 10.1038/s41598-018-36488-9 (PMC6344545; doi:10.1038/s41598-018-36488-9)
Supplement: Supplementary file 1 — Supplementary information [file 41598_2018_36488_MOESM1_ESM.pdf]

Quorum sensing in *Pseudomonas aeruginosa* mediated by RhlR is regulated by a small RNA PhrD

Anuja Malgaonkar[1] and Mrinalini Nair[1*]

[1]Department of Microbiology and Biotechnology Centre, The Maharaja Sayajirao University of Baroda, Vadodara, Gujarat, India.

Correspondence:

Mrinalini Nair
mnair_in@yahoo.com
Telephone no: +91-265-2794396
Fax: +91-265-2792508

Table S1 List of putative targets of PhrD in RNA Predator program

Rank	Energy (kJ/mole)	Z score	Targets
1	-21.53	-4.67	major facilitator transporter
2	-21.22	-4.56	TolR protein
3	-20.54	-4.32	putative transporter
4	-19.76	-4.04	hypothetical protein PA2935
5	-19.52	-3.95	hypothetical protein PA4536
6	-19.47	-3.93	Transposase
7	-19.39	-3.9	hypothetical protein PA2777
8	-19.38	-3.9	Hcp1
9	-19.24	-3.85	hypothetical protein PA2126
10	-19.12	-3.8	DNA polymerase III subunit alpha
11	-18.98	-3.75	4 hydroxy 3 methylbut 2 enyl diphosphate reductase
12	-18.87	-3.71	hypothetical protein PA4712
13	-18.44	-3.56	hypothetical protein PA0819
14	-18.37	-3.53	hypothetical protein PA5132
15	-18.28	-3.5	hypothetical protein PA4685
16	-17.99	-3.4	putative serine/threonine protein kinase
17	-17.99	-3.4	putative glycosyl transferase
18	-17.88	-3.36	hypothetical protein PA2462
19	-17.87	-3.35	putative glutathione peroxidise
20	-17.74	-3.31	chemotaxis specific methylesterase
21	-17.72	-3.3	hemin importer ATP binding subunit
22	-17.69	-3.29	apolipoprotein N acyltransferase
23	-17.68	-3.29	NADH dehydrogenase subunit N
24	-17.66	-3.28	putative aminopeptidase
25	-17.62	-3.26	type III secretion system protein
26	-17.59	-3.25	hypothetical protein PA2222
27	-17.54	-3.23	pyrroloquinoline quinone biosynthesis protein F
28	-17.53	-3.23	NosY protein
29	-17.48	-3.21	multidrug efflux protein NorA
30	-17.46	-3.21	GbdR
31	-17.32	-3.16	ClpB protein
32	-17.28	-3.14	arginyl tRNA synthetase
33	-17.10	-3.08	branched chain amino acid ABC transporter permease
34	-17.08	-3.07	hypothetical protein PA1111
35	-17.02	-3.05	putative iron sulfur cluster binding protein; Rieske family
36	-16.92	-3.01	PvdJ
37	-16.89	-3	transcriptional regulator RhlR
38	-16.81	-2.97	'two component response regulator; CopR '
39	-16.81	-2.97	4 amino 4 deoxy L arabinose transferase
40	-16.76	-2.95	hypothetical protein PA2502
41	-16.69	-2.93	hypothetical protein PA4612
42	-16.60	-2.9	ABC transporter permease
43	-16.56	-2.88	hypothetical protein PA3127
44	-16.55	-2.88	putative oxidoreductase
45	-16.51	-2.86	major facilitator transporter
46	-16.45	-2.84	BifA

47	-16.44	-2.84	putative transcriptional regulator
48	-16.38	-2.82	transcriptional regulator
49	-16.26	-2.77	manganese transport protein MntH
50	-16.22	-2.76	hypothetical protein PA2984

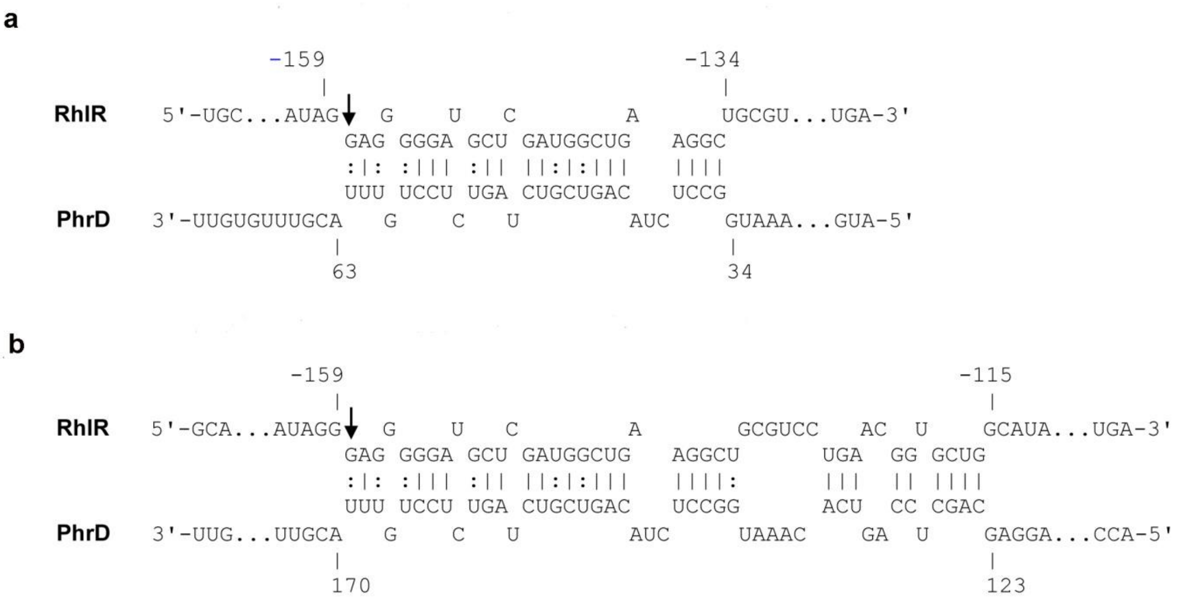

Figure S1 Comparison of RNA-RNA interaction of rhlR with PhrD expressed from the chromosome (a) and plasmid (b). The former interaction is completely seen in the later as indicated by IntaRNA program. The numbers on PhrD indicate nucleotide from the transcription start site.

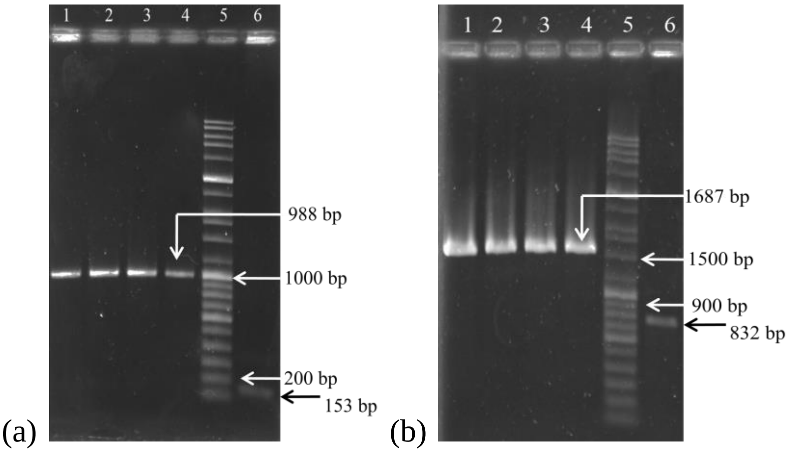

Figure S2 Confirmation of *phrD* disruption by PCR. **(a)** PhrD ncRNA F/R primers; Lane 1-4: Double cross over *phrD* mutants, Lane 5: High range marker (100-10,000 bp), Lane 6: PAO1 WT (amplicon of 153bp from WT PhrD) **(b)** PhrD ncRNA F/ PhrD Dn R primers; Lane 1-4: Double cross over *phrD* mutants, Lane 5: High range marker (100-10,000 bp), Lane 6: PAO1 WT **(c)** Gm F/ PhrD Dn R primers; Lane 1-4: Double cross over mutants, Lane 5: High range marker (100-10,000 bp), Lane 6: PAO1 WT (no amplification due to absence of gentamicin casette).

(a) 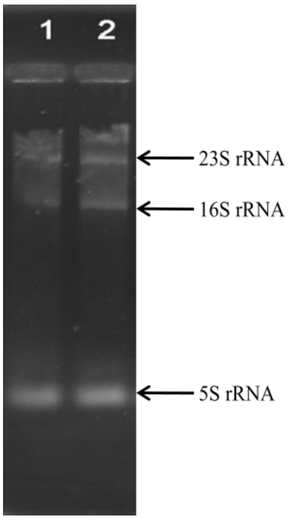

(b) 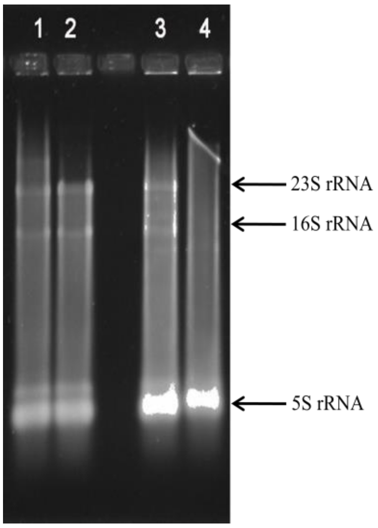

Figure S3 RNA for northern blot of *phrD* expression under different nutrient conditions. **(a)** Lane 1 and 2: RNA extracted from cells grown in PPGAS medium. **(b)** Lane 1 and 2: RNA extracted from cells grown in Luria broth, Lane 3 and 4: RNA extracted from cells grown in MMP medium.
